# The ripple effect of strain in times of change: how manager emotional exhaustion affects team psychological safety and readiness to change

**DOI:** 10.3389/fpsyg.2024.1298104

**Published:** 2024-03-11

**Authors:** Patrick Groulx, Francis Maisonneuve, Jean-François Harvey, Kevin J. Johnson

**Affiliations:** ^1^Department of Management, HEC Montreal, Montreal, QC, Canada; ^2^Department of Human Resource Management, HEC Montreal, Montreal, QC, Canada; ^3^Department of Entrepreneurship and Innovation, HEC Montreal, Montreal, QC, Canada

**Keywords:** manager’s emotional exhaustion, *laissez-faire* leadership, team readiness to change, psychological safety, public administration

## Abstract

**Introduction:**

Managers assume a pivotal role during periods of organizational change, yet there exists a notable gap in our understanding of how their emotional exhaustion may impact their capacity to generate readiness to change within their teams. Grounded in the conservation of resources theory (COR), this study explores the crossover effect of managers’ emotional exhaustion on team readiness to change. We expect this to occur through higher levels of *laissez-faire* leadership, which impacts the teams’ psychological safety.

**Methodology:**

Data was gathered within a Canadian governmental organization undergoing two significant changes—cultural change and digitalization—with a specific focus on leadership as a pivotal factor in preparing teams for change. Employing surveys from 372 team members and 62 managers affected by this change, we conducted path analysis to empirically test the proposed model across 74 teams and their respective managers.

**Results:**

Managers’ emotional exhaustion has a negative indirect effect on team readiness to change. The double mediation pathway implies a positive relationship on *laissez-faire* leadership, which hinders psychological safety. In turn, psychological safety hampers team readiness to change.

**Conclusion:**

Managers must invest significant resources to fulfill their roles and responsibilities during strategic change. Those who feel exhausted during change may look for ways to protect some of their resources by reducing the time and energy they invest leading their team. This self-preserving resource strategy has detrimental consequences on teams’ effectiveness during change due to an indirect crossover effect that affects the levels of psychological safety on the team.

## 1 Introduction

The role of managers’ leadership in supporting their teams through organizational change has garnered significant attention (Decoster et al., 2023; Harvey and Kudesia, 2023; Potosky and Azan, 2023). Research shows that certain types of leadership (e.g., transformational leadership) enhance followers’ attitudes and behavior during change (Eisenbach et al., 1999; Faupel and Süß, 2019; Oreg and Berson, 2019; Potosky and Azan, 2023). Other scholars have looked at the dynamic managerial capabilities of managers throughout the organization, emphasizing their cognitive makeup (Helfat and Peteraf, 2015; Harvey and Kudesia, 2023). They have shown, for instance, that managers’ mindful attention can stimulate experimentation in teams and make them more receptive to change.

However, research has scantly considered managers’ psychological resources during change, despite the fact that they are likely to influence teams’ adaptive capabilities (Oreg and Berson, 2019). Specifically, managers are at risk of experiencing emotional exhaustion during change due to the increase of their roles and responsibilities (Balogun and Johnson, 2004; Decoster et al., 2023), and the heightened ambiguity they grapple with (Bordia et al., 2004; Johnson, 2016; Harvey and Kudesia, 2023). Managers who are lacking psychological resources may be less suited to invest time and energy into leadership behavior (Hobfoll et al., 2018), and these likely limit teams’ capabilities to change. These relationships have yet to be tested empirically, and the pathway through which managers’ psychological resources, or lack thereof, affect teams’ capabilities for change remains a black box.

**Figure 1:**
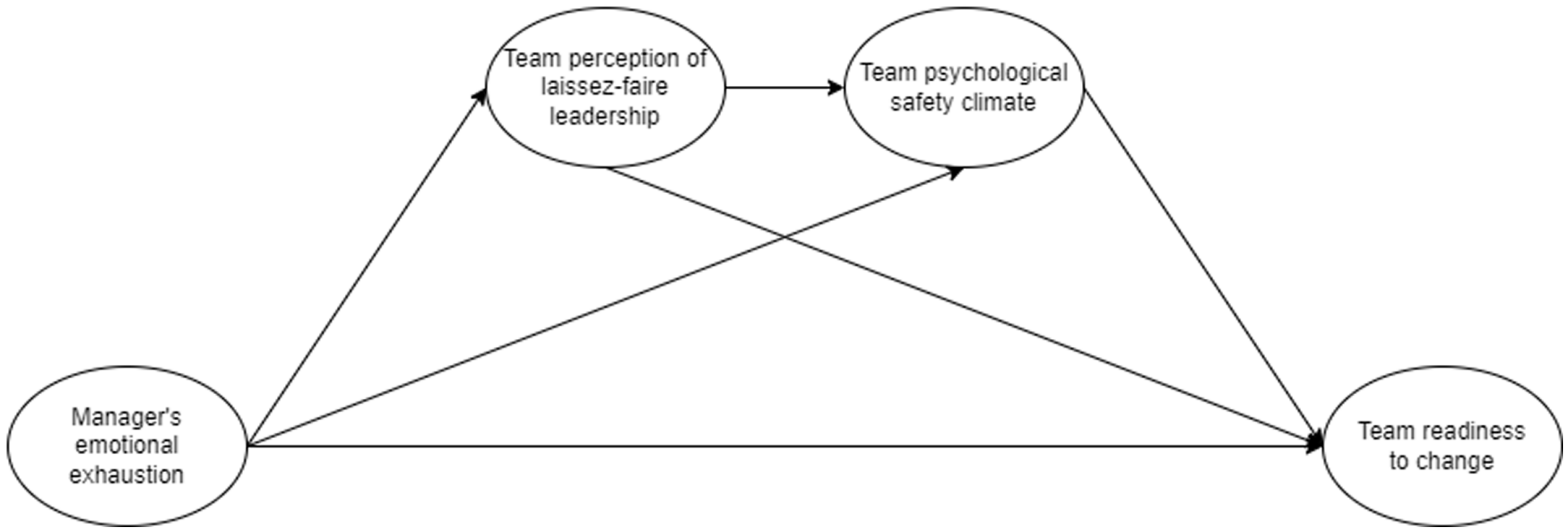
Theoretical model.

Building on the conservation of resource theory (COR) and its principle of resources crossover (Hobfoll et al., 2018), we suggest a path through which managers’ resource scarcity impacts teams’ collective attitude towards change. As depicted in Figure 1, we argue that to protect their remaining resources (Halbesleben et al., 2014), emotionally exhausted managers tend to neglect their leadership responsibilities—adopting a leadership style that is less demanding such as *laissez-faire*. Defined as the “absence of leadership, the avoidance of intervention, or both” (Bass and Avolio, 1990, p. 20), *laissez-faire* leadership can affect the teams’ climate. In particular, we argue that this type of leadership hinders psychological safety, or the feeling that the team is safe for interpersonal risk taking such as asking for help, asking questions, and experimenting (Edmondson, 1999; Harvey et al., 2019). Psychologically safe teams can better develop their change capabilities (Edmondson et al., 2001). However, to maintain high levels of psychological safety, teams going through strategic change likely need supportive managers.

Our study contributes to the literature in three ways. First, we uncover the often-overlooked phenomenon of managers’ indirect resource crossover effects, revealing their pivotal role in shaping team emergent states. This extends current studies looking at the dyadic effects of resource crossover (Mesmer-Magnus and DeChurch, 2009; Wang et al., 2010; Huang et al., 2016) or how team resources transfer to individuals (Chênevert et al., 2019). This study also joins recent efforts of considering managers as change recipients as well as change agents (Decoster et al., 2023). It opens up new opportunities for research to explore the influence of managers beyond their leadership style. Second, our study deepens our understanding regarding the adoption of a laisse-faire leadership style. Adopting a psychological resource perspective, we suggest that managers adopt *laissez-faire* leadership as a self-preserving behavior, rather than incompetence as suggested in the literature (Skogstad et al., 2007; Breevaart and Zacher, 2019). By doing so, we also answer the call for identifying additional antecedents and outcomes of *laissez-faire* leadership (Robert and Vandenberghe, 2022). Third, our study clarifies the relationship between *laissez-faire* leadership and psychological safety in the context of organizational change. Previous research suggested that *laissez-faire* leadership could be beneficial for psychological safety. Our research shows that, in the context of change, *laissez-faire* leadership has a negative impact on teams. Additionally, this paper joins recent efforts of conceptualizing change reaction at the team-level (Groulx et al., 2023; Harvey and Kudesia, 2023). Despite recognition that change is a group phenomenon (Bouckenooghe, 2010; Harvey et al., 2022), most studies adopted an individual perspective on change.

## 2 Theoretical basis and literature review

### 2.1 COR theory and the concept of crossover

The conservation of resource theory (COR) is a motivational theory which explains and predicts individuals’ behaviors based on the availability of their resource pool (Chen et al., 2015). According to COR, individuals strive to obtain, retain, foster, and protect the resources they value (Hobfoll, 1989). Based on this tenet, scholars propose that individuals in situations of resource scarcity (e.g., when they are emotionally exhausted) tend to reduce their efforts and their resource investment towards answering job demands (Hobfoll, 1989). In such a condition, individuals tend to conserve the remaining resources as a survival function by investing in more self-preserving mechanisms, and therefore avoid the risk of experiencing an additional resource loss spiral.

Crossover effects are defined as “the interpersonal process that occurs when the job stress or psychological strain experienced by one person affects the level of strain of another person in the same social environment” (Hobfoll et al., 2018, p. 108). It accounts for resource transfers between members of a given social system, such as managers and their employees (Chen et al., 2015). Westman (2001) suggested three underlying mechanisms through which the crossover process may occur: empathy (direct crossover), mediating or moderating mechanisms (indirect crossover), or sharing common stressors (common crossover).

In this article, we focus on indirect crossover effects, or the distinctive mediating mechanisms that transmit experiences (Westman, 2001), specifically, in the context of organizational change. More precisely, we argue that as managers experience higher levels of resource scarcity, they will invest less effort towards their role and responsibilities within their team. As such, team members will perceive this absence of action as *laissez-faire* leadership, which will affect the establishment of a psychological safety climate, which is known to be important during organizational change (Edmondson and Bransby, 2023). The theoretical model is presented in Figure 1.

## 3 Hypotheses development

### 3.1 *Laissez-faire* leadership as a self-preserving behavior for leaders

Managers play a predominant role in shaping team readiness to change through their influence over team processes and outcomes during organizational change (Armenakis and Harris, 2002). They can do so by embracing experimentation in their own practice (Harvey and Kudesia, 2023), and framing the team’s work as a learning project (Edmondson et al., 2001). They can also provide constructive feedback (Harvey and Green, 2022), and reshuffle team membership to mix new and old perspectives (Groulx et al., 2023).

However, these actions require significant resource investments from the managers (e.g., time, physical energy, emotional energy, attention). The perception of available resources has an important impact on individuals’ decisions and behaviors, regardless of the objective situation (Clarkson et al., 2010; Halbesleben et al., 2014). Managers who feel depleted from their resources are known to experience higher levels of emotional exhaustion (Hobfoll et al., 2018). Scholars show that managers with higher levels of emotional exhaustion have limited resources to accomplish leadership tasks (Wright and Cropanzano, 1998; Maslach et al., 2001) and focus their energy on different ways to defend their remaining resource pool (Halbesleben et al., 2014). These resource protection strategies aim to protect them from further resource depletion (Hobfoll et al., 2018) at the cost of restricting their involvement and protection towards other stakeholders, including their own team.

As managers with scarce resources adopt defensive strategies to protect their remaining resources (Halbesleben et al., 2014), they can impoverish the quality and quantity of their interactions with their team members, resulting in a weaker leader-member relationship. As such, teams’ needs and expectations may not be met, which can increase their perception of *laissez-faire* leadership. Altogether, we hypothesize that:

*H1*: Managers’ emotional exhaustion is positively related to their *laissez-faire* leadership.

### 3.2 From *laissez-faire* leadership to psychological safety

Scholars argue that certain team emergent states develop in reaction to team leadership (Harvey et al., 2019; Mao et al., 2019). Specifically, since the foundational work from Edmondson (1999), the relationship between leadership and psychological safety has been demonstrated in a variety of settings (e.g., Cannon and Edmondson, 2001; Day et al., 2004; Nembhard and Edmondson, 2006; Edmondson and Harvey, 2017, 2018). Scholars argue the presence of competent managers increases the perception of a psychological safety climate, as they reduce the anxiety of their team members (Mao et al., 2019). Such actions signal to team members that their manager is a credible source of support, help, and guidance. Other scholars also suggest that proactive behaviors such as inviting team members to provide feedback in crucial moments (Ortega et al., 2014; Edmondson and Harvey, 2018; Coutifaris and Grant, 2022), listening to team members’ concerns (Castro et al., 2018), and demonstrating competency and transparency (Yi et al., 2017) have beneficial effects on teams’ perception of psychological safety.

As such, *laissez-faire* leadership may produce the opposite effect by increasing the adversity in teams (Neves and Schyns, 2018; Otto et al., 2018). Indeed, the neglect of managers may deprive teams of significant resources (Robert and Vandenberghe, 2022; Edmondson and Bransby, 2023) that enable the development of psychological safety. In addition, it may nurture important stressors (e.g., role ambiguity, cynicism, co-worker conflicts) which can also negatively impact levels of psychological safety on the team. For these reasons, we argue that:

*H2*: *Laissez-faire* leadership is negatively related to team psychological safety.

### 3.3 From psychological safety to readiness to change

Drawing from Groulx et al. (2023:4), team readiness to change captures “members’ beliefs, attitudes, and intentions concerning the necessity of changes and the organization’s capability to effectively implement those changes”. It develops through the emergence of individuals’ cognition and emotions which become shared through social interaction processes (Rafferty et al., 2013; Groulx et al., 2023). As team members interact about the common stimuli (e.g., top-down processes, leadership, organizational events), a consensual view about their level of readiness to change can emerge. Communications about a change vision that explicitly addresses what the change means for the team can facilitate its emergence (Rafferty et al., 2013). Prior research indicates that specific change-related beliefs, including discrepancy, appropriateness, change self-efficacy, principal support, and valence, serve as the closest predictors of an individual’s readiness to change (Armenakis et al., 1993, 2007; Armenakis and Harris, 2002; Rafferty and Minbashian, 2019). Recent research has also underscored the significance of emotions in this context as well (see Oreg and Berson, 2019; Rafferty and Minbashian, 2019). Nevertheless, studies looking at team reactions to change remain limited (Groulx et al., 2023; Harvey and Kudesia, 2023). Groulx et al. (2023) demonstrated that team processes, such as team reflexivity, can positively influence team-level readiness for change. Additionally, Chênevert et al. (2019) proposed that team support plays a pivotal role in generating individual readiness to change.

Building on these findings, we argue that team readiness to change is most likely to be facilitated by team psychological safety. Being ready to change requires teams to be willing to adopt new processes and voice concerns when needed (Nembhard and Edmondson, 2006; Newman et al., 2017; Edmondson and Bransby, 2023). Team members to engage in learning behaviors such as experimentation, trial and error and seeking help to find solutions to new problems (Edmondson et al., 2001; Singer et al., 2015; Harvey and Kudesia, 2023). Part of adopting new procedures also include receiving and giving feedback to team members (Nembhard and Edmondson, 2006; Harvey and Green, 2022). Such behaviors involve a certain personal and interpersonal risk. As such, feeling that it is safe to express and share ideas without fear of negative consequences may encourage mature and open discussions about the change initiatives (Rafferty et al., 2013). Simultaneously, this can also facilitate upward-directed communication and voicing of employees’ concerns, which is known to have positive impacts on change-related attitudes (Edmondson and Lei, 2014; Edmondson and Bransby, 2023). Overall, constructive communications among team members facilitated by psychological safety can nurture team-level readiness to change (Rafferty and Jimmieson, 2010; Rafferty et al., 2013). We therefore argue that:

*H3*: Team psychological safety is positively related to team readiness to change.

Altogether, we argue that when managers are in a condition of resource scarcity— or emotionally exhausted—they tend to reduce their resource investment within their work (Hobfoll et al., 2018). This will have an indirect crossover effect on teams’ perception of readiness to change, as members will increasingly perceive the leadership style as *laissez-faire*, which harms the development of a psychological safety climate. In sum:

*H4*: The relationship between managers’ emotional exhaustion and team readiness to change is sequentially mediated by *laissez-faire* leadership and team psychological safety, such that managers experiencing higher levels of emotional exhaustion are more likely to exhibit *laissez-faire* leadership behaviors, which, in turn, diminish team psychological safety, ultimately hindering team readiness to change.

## 4 Methods

### 4.1 Sample and procedure

This study took place in a Canadian governmental organization where employees were experiencing a major organizational transformation in 2019. The goal of this transformation was twofold. First, they wanted to digitize certain provided services and transfer others onto a cloud server. Second, the organization was going through a cultural change towards a more participative and collaborative organization and wanted to flatten its structure to optimize customer service. Practices such as implementation of a matrix structure, establishment of coordination practices, and review of key performance indicators were planned during this transformation. Employees mainly worked in teams that developed financial products for the population. Participants were selected based on a list of existing teams provided by the HR department. All managers of these teams were also solicited to answer this survey.

Using corporate email addresses, we sent out 569 surveys to respondents across 98 teams. The first page of the survey informed the participants about research ethics and response confidentiality. This survey was used by the organization to diagnose their change capacity and identify which team and/or department needed additional support during this transformation. To assure confidentiality of members’ response, teams with small sample size (lower than 6) were not reported to the organization. To optimize participation, one email was sent per week to employees who did not complete their surveys. We received a total of 449 (78.91%) valid and completed surveys. From these surveys, we selected the teams for which we had the full survey of the manager and for which 40% of the employees also responded. In total, we kept 372 employees (65%), 74 teams (75.51%; manager and non-management teams), and 62 managers (some managers were managing two teams) with an average size of 7.28 employees per team and team tenure of 2 years.

### 4.2 Measures

#### 4.2.1 Manager reported measure

##### 4.2.1.1 Emotional exhaustion

Managers had to complete the emotional exhaustion scale developed by Maslach et al. (1997). It is composed of 7 items on a 7-point Likert scale. Sample item is: “I feel emotionally drained from my work” (α = 0.91).

#### 4.2.2 Team reported measures

##### 4.2.2.1 *Laissez-faire* leadership

We used the *laissez-faire* leadership scale by Bass and Avolio’s (2000). It is composed of 4 items rated on the frequency of behavior (1 = never; 5 = always). Sample item is: “My supervisor avoids getting involved when important issues arise” (α = 0.91).

##### 4.2.2.2 Psychological safety

We used Harvey et al. (2019) short scale of psychological safety. It is composed of 4 items rated on a 7-point Likert scale. Sample item is: “In this team, it is easy to speak up about what is on your mind” (α = 0.87).

##### 4.2.2.3 Team readiness to change

We used Groulx et al. (2023) team readiness to change scale. It is composed of 4 items on a 7-point Likert scale. Participants were asked to give their answers in relation to their team. Sample items are “We are ready for these organizational changes” and “We would consider ourselves open to these changes” (α = 0.95).

#### 4.2.3 Control variables

##### 4.2.3.1 Team size

Team size was controlled in our model due to previous studies showing a negative relationship with team adaptation related variables (Schippers et al., 2015), but positively related to team innovation (Hülsheger et al., 2009).

##### 4.2.3.2 Team tenure

We controlled for team tenure given that Groulx et al. (2023) showed its effect on team readiness to change. Team tenure was assessed using tenure brackets in increments of 6 months (except for the first bracket of 0–3 months and the last bracket of 34 months and more). Participants were asked to indicate how long they were assigned to their specific current team. We then computed the team tenure using the average team members’ tenure.

### 4.3 Validity evidence

#### 4.3.1 Confirmatory factor analysis

We performed a confirmatory factor analysis (CFA) to confirm the validity and the distinctiveness of each latent variable. We modelled our four latent variables, each observed through their respective items while allowing covariation between latent variables. Our results suggest a satisfactory structure (*χ*^2^ = 185.66; *df* = 146; CFI = 0.97; TLI = 0.96, RMSEA = 0.061; SRMR = 0.078). All observed variable loaded to their respective latent variable (min = 0.48, max = 0.98). We then compared our theorized model to three other alternate models. All fit indices and chi-square difference tests show that our model provides the best fit for the data (Table 1).

**Table 1:** Comparison of alternative models.

Models	*χ* ^2^	*df*	CFI	TLI	RMSEA	SRMR	*Δ χ* ^2^
4-Factor model	185.66	146	0.97	0.96	0.06	0.08	
3-Factor model psychological safety-team readiness to change	303.54	149	0.87	0.85	0.12	0.10	^**^
3-Factor model *laissez-faire*-Manager emotional exhaustion	392.53	149	0.80	0.77	0.15	0.18	^**^
1 Factor model	797.87	152	0.46	0.39	0.24	0.23	^**^

^*^p < 0.05, ^**^p < 0.01. 3-Factor model psychological safety-team readiness to change: consists of a model where the team readiness to change and psychological safety were modelled under the same latent factor; 3-factor model laissez-faire-manager emotional exhaustion: consists of a model where laissez-faire and managers’ self-report measurement of emotional exhaustion were modelled under the same latent factor.

#### 4.3.2 Data aggregation

To demonstrate sufficient within-group and between-group heterogeneity, we computed the rwg (j), ICC (1), and ICC (2) for each variable (Chen and Bliese, 2002). According to LeBreton and Senter’s (2008) cut-off criteria, we obtained a strong agreement for *laissez-faire* leadership (rwg (j) = 0.86, SD = 0.24), readiness to change (rwg (j) = 0.84, SD = 0.21), and for psychological safety (rwg (j) = 0.81, SD = 0.24).

All ICC (1) scores were > 0, and the associated One-Way ANOVA analyses were all significant at *p* < 0.05. As for the ICC (2), we obtained 0.52 for *laissez-faire* leadership, 0.59 for readiness to change, 0.52 for the meaning sub-dimension of empowerment, and 0.036 for psychological safety. Although the suggested cut-off criterion of ICC (2) is set at 0.60 by Glick (1985), many scholars argue that this is an arbitrary criterion (LeBreton and Senter, 2008; Harvey et al., 2019). Other scholars argue that values >0.25 are acceptable when the rwg (j) is high and when the ICC (1) and its *F*-test results have met the criterion. We therefore proceeded to aggregate our data.

#### 4.3.3 Convergent and discriminant validity

To assess convergent validity, we first computed all composite reliability (CR) factors. All CR were higher than the suggested cut-off of 0.80 (Netemeyer et al., 2003). We computed the average variance extracted (AVE) for emotional exhaustion (0.59), *laissez-faire* leadership (0.73), psychological safety (0.67), and for team readiness to change (0.83) and they were all higher than the cut-off criterion of 0.50. As for the discriminant validity, all AVE indices were higher than each factor’s maximum shared variance (MSV). In sum, these results support convergent and discriminant validity.

## 5 Hypotheses testing and results

We tested the model using Hayes (2013) two mediation model (PROCESS 3.5, model 6). We also provided a bootstrap analysis (95%, sample = 5,000) to estimate the different indirect effects underlying this model.

Means, standard deviations, and bivariate correlations for all variables are presented in Table 2. Results of our theorized model suggest good fit with control variables (*χ*^2^ = 221.06; *df* = 176; CFI = 0.96; TLI = 0.96, RMSEA = 0.059; SRMR = 0.075). Many strong relationships were found. Psychological safety and team readiness to change were highly correlated (*r* = 0.65, *p* < 0.01), despite showing discriminant validity. *Laissez-faire* leadership was also correlated to team readiness to change (*r* = −0.44, *p* < 0.01), and with psychological safety (*r* = −0.44, *p* < 0.01). It is also noteworthy to mention that team size was negatively correlated to team readiness to change (*r* = −0.23, *p* < 0.05,) and marginally significantly correlated to team psychological safety (*r* = −0.21, *p* < 0.10), thus supporting its inclusion in the model as a control variable.

**Table 2:** Means, standard deviations, correlations, and reliabilities of studied variables.

Variables	*M*	*SD*	1	2	3	4	5	6
1. Team size	7.28	3.45	–					
2. Team tenure	2.00	1.23	−0.07	–				
3. Manager’s emotional exhaustion	2.94	1.34	0.11	−0.2	(0.91)			
4. Leadership *laissez-faire*	1.47	0.43	−0.12	−0.19	0.31^**^	(0.91)		
5. Psychological Safety	5.47	0.66	−0.21^†^	0.09	−0.17	−0.44^**^	(0.87)	
6. Team readiness to change	4.96	0.79	−0.23^*^	0.02	−0.26^*^	−0.44^**^	0.65^**^	(0.95)

*N* = 74. ^†^*p* < 0.10, ^*^p < 0.05, ^**^p < 0.01 (two-tailed).

*Hypothesis 1* suggested that a manager’s level of emotional exhaustion was positively related to their team’s perception of *laissez-faire* leadership. The observed relationship was significant and in the proposed direction (*β* = 0.32, *p* < 0.01), lending support to *Hypothesis 1*. *Hypothesis 2* posited that teams’ perception of *laissez-faire* leadership was negatively related to team psychological safety. The relationship was significant and in the proposed direction (*β* = −0.48, *p* < 0.01), supporting *Hypothesis 2*. Of note, team size was also negatively related to psychological safety (*β* = −0.27, *p* < 0.05), suggesting that as team size increases, team members perceive less psychological safety. It was the only occurrence of a significant relationship regarding control variables. *Hypothesis 3* suggested that team psychological safety was positively related to team readiness to change. Again, the relationship was significant and in the proposed direction (*β* = 0.52, *p* < 0.01), supporting *Hypothesis 3*.

Finally, *Hypothesis 4* suggested a full mediation model, whereas the relationship between a manager’s level of emotional exhaustion and team readiness to change was mediated by the team’s perception of *laissez-faire* leadership, and in turn by the team psychological safety. The 5,000-bootstrap sample analysis with a 95% confidence interval demonstrated that the negative total effect of managers’ emotional exhaustion on team readiness to change was significant (estimate effect = −0.14, SE = 0.07, *t* = −0.2010, *p* = 0.04). The completely standardized indirect effect of managers’ level of emotional exhaustion on team readiness to change was also significative (*γ* = −0.08, BootSE = 0.04, LLCI: −0.1706, ULCI: −0.0118). In addition, the indirect effect of managers’ level of emotional exhaustion on team readiness to change through *laissez-faire* leadership was also significative (*γ* = −0.04, BootSE = 0.03, LLCI: −0.1743, ULCI: −0.0063). Overall, these results supported *Hypothesis 4*. The results regarding the full model are presented in Table 3.

**Table 3:** Results of the full model.

Variables	*Laissez-faire* leadership		Team psychological safety		Team readiness to change	
	*β*	*p*-value	*β*	*p*-value	*β*	*p*-value
*Control variable*						
Team size	−0.16	0.14	−0.27	0.01	−0.15	0.12
Team tenure	−0.20	0.07	−0.03	0.83	−0.08	0.35
*Principal effects*						
Manager’s emotional exhaustion	0.32	0.01	0.00	0.98	−0.09	0.33
*Laissez-faire* leadership			−0.48	0.00	−0.22	0.04
Team psychological safety					0.52	0.00
*R* ^2^	0.16		0.25		0.49	

## 6 Discussion

Given that organizational change is a demanding task for managers, our study aimed at demonstrating the crossover effect of managers’ strain on teams’ collective attitude towards change. Managers act as change agents, translating strategic objectives into actionable plans and motivating their teams to adapt to change (Harvey and Kudesia, 2023). The demanding nature of leadership during change places a considerable burden on managers, exposing them to heightened stressors and challenges, particularly in the context of substantial organizational change such as digitalization and cultural change, as observed in our study. We drew on COR theory (Hobfoll, 1989; Hobfoll et al., 2018) to consider the psychological distress of managers and develop theory on how it can impact team dynamics. Specifically, we theorized that managers with scarce psychological resources would adopt defensive strategies to prevent additional psychological resources loss, translating into laissez-faire leadership. Such leadership would then affect teams’ readiness to change by negatively impacting the psychological safety climate.

### 6.1 Theoretical implications

Our study makes three significant contributions. First, we shed light on the influential role of indirect resource crossover effects in shaping the development of team emergent states. While prior research has predominantly focused on direct resource transfers among team members (Mesmer-Magnus and DeChurch, 2009; Wang et al., 2010), examined dyadic leader-member relationships (Huang et al., 2016), or investigated individual perceptions of crossover (Chênevert et al., 2019), our approach delves deeper into the intricate mechanics of how managers’ depletion of psychological resources can indirectly impact teams and influence the development of emergent states. Consequently, we show that the effects of resource crossover extend beyond individual reactions, stretching into the realm of collective responses to change. This research thereby contributes to the growing body of work that underscores the critical importance of understanding change-related concepts within the context of teams (Harvey and Kudesia, 2023). Furthermore, our work answers the call from COR scholars for further exploration of crossover effects within teams (Hobfoll et al., 2018) and aligns with the broader movement towards applying COR principles to team dynamics (Stoverink et al., 2020).

Second, this article also contributes to the leadership literature by adopting a resource perspective. We know that passive leadership styles such as *laissez-faire* leadership is prevalent in organizations and has a negative impact on employees’ well-being (Lundmark et al., 2022; Robert and Vandenberghe, 2022), change outcomes (Bligh et al., 2018; Lundmark et al., 2022), and role ambiguity (Skogstad et al., 2014). Up to now, research has focused mainly on the consequences of different types of leadership (Skogstad et al., 2007; Robert and Vandenberghe, 2022) rather than looking at individual differences and reasons why managers adopt one particular approach. We do so by building on the COR theory (Hobfoll, 1989; Hobfoll et al., 2018) and suggesting that managers’ roles and responsibilities require substantial investments towards their team. Consequently, when managers are exhausted, they may avoid their role and responsibilities, not because of incompetency, but rather as a self-preserving mechanism to avoid additional resource loss. As such, these findings are important because it changes our perspective as to how to deal with such leadership approach in organizations. *Laissez-faire* behavior is not only a question of skill acquisition and development, but also of supporting managers’ psychological resources in times of high demands. This contribution provides new avenues to research regarding managers’ resources and their leadership style. Therefore, we invite other leadership scholars to explore the cost of having proactive leadership behaviors and how they impact the physical and psychological well-being of leaders. This resource perspective towards leadership also opens new avenue of research as to how managers’ psychological resources complement managerial dynamic capabilities such as social capital, managerial cognition, human capital, and emotional capabilities during change (Helfat and Peteraf, 2015; Harvey and Kudesia, 2023). Following our results, we would suspect that managers’ psychological resources should act as enablers of these dynamic capabilities.

Lastly, this study also provides a contribution by elucidating the relationship between *laissez-faire* leadership and psychological safety in the context of organizational change. Prior research has hinted at the potential benefits of *laissez-faire* leadership for outcomes such as innovation propensity (Ryan and Tipu, 2013; Yang, 2015). Here, we focus specifically on organizational change, a situation where managers play a pivotal role in translating strategic intentions into actionable steps. Scholars have contended that excessive leadership involvement might hinder motivational advantages compared to situations where team members collaboratively negotiate and determine the group’s regulatory actions (Panadero et al., 2015). Others have suggested parallels between *laissez-faire* leadership and empowering leadership, as both involve granting higher levels of decision-making participation and responsibilities to team members (Wong and Giessner, 2018). Nonetheless, it’s essential to recognize that *laissez-faire* leadership primarily entails relinquishing managerial responsibilities due to a lack of psychological resources. In contrast, empowerment centers on recognizing and appreciating team members’ skills and autonomy (Wong and Giessner, 2018). This requires managers to invest a significant amount of time and energy to structure their team and to establish a proper team climate for such an emergent state to develop.

### 6.2 Practical implications

Knowing that strategic resources are limited within organizations, especially during organizational change, our study suggests that investing in the well-being of managers can have a positive crossover effect on their teams’ capacity to change. Providing resources to managers can allow them to invest into proactive leadership behavior without succumbing to exhaustion. For instance, being accompanied by organizational development specialists can also limit the stressful aspect of change for managers. The expertise of specialists is valuable in supporting managers when their team is under pressure. Furthermore, organizations may offer a variety of training before change occurs to ensure that managers have the right tools to effectively manage change in their teams. Results from our study indicate that it’s not only a matter of competency, but mainly a question of resources. As such, we recommend organizations to be cautious regarding the amount of training that managers must go through before and during the change process. Indeed, as much as training enable managers to gain additional resources in terms of competency, it also requires energy and time to accomplish them. Consequently, building a training program that answers their specific needs may be more impactful.

Our study also stresses the importance of managers establishing a psychological safety climate in their teams during organizational change process. As such, encouraging open communication where employees feel comfortable speaking up and sharing their constructive criticism can be very useful for teams and the organization. Proactive behaviors such as addressing conflicts and concerns in teams before they get out of hand can support the emergence of psychological safety climate in teams.

Finally, our results also suggest that the size of teams can impact their perception regarding readiness to change. Indeed, coordinating a greater number of employees, assuring that they hold a common vision of the change process, and responding to their needs can be difficult for managers. Thus, we suggest that organizations should structure teams as smaller units to ease the development of readiness to change.

### 6.3 Limitations and future studies

Despite the constructive implications of our study, no study is without limitation. First, our study would gain from replication in different settings since it took place in a governmental organization. These organizations are more rigid and hierarchical, which implies that their employees are more dependent on their managers to obtain change-related information. Future studies could compare how the power distance or level of bureaucracy impacts the importance of the managers’ role in generating readiness to change. In addition, comparing our results with a sample of teams in private organizations could help test the generalization of the findings. Private organizations are often less bureaucratic where information flows more freely between levels and within teams. As such, each individual team member is less dependent on their manager’s initiative regarding change management. Furthermore, exploring the difference between various forms of teams could provide more fine-tuned results. Occurrence of temporary teams is increasing as matrix-based work structure becomes more prevalent, which could alter how team emergent states develop. The autonomous work team is another form that is gaining in popularity which could impact the magnitude of the relationship between leadership style and team emergent states.

Second, our research focused on *laissez-faire*, measuring no other leadership style. Future research should inquire regarding potential variations in the effect of managers’ emotional exhaustion on other leadership style adopted. Perhaps certain managers would be eager to become transactional leaders, while others may seek to micromanage or delegate to prevent further resource loss. Others could continue overinvesting into their team, hoping to receive resources from reciprocating team members. As such, boundary conditions, such as personality traits or leadership-member exchange, could open different avenues of future research to develop a better understanding of the resource investment strategies pursued by exhausted managers. In addition, future research should also delve into the long-term consequences of *laissez-faire* leadership on leaders’ health and well-being. While *laissez-faire* leadership may initially serve as a self-preserving mechanism for managers, exploring its sustained impact over time is crucial. Longitudinal studies can investigate the evolving dynamics between *laissez-faire* leadership, team productivity, resistance to change, and psychological safety, shedding light on the reciprocal relationships that may contribute to the gradual depletion of managers’ psychological resources.

Third, while our study establishes a connection between managers’ emotional exhaustion and their teams’ perception of *laissez-faire* leadership, it is imperative to delve deeper into the repertoire of coping mechanisms employed by managers undergoing organizational changes. Beyond neglecting team responsibilities, some managers may adopt alternative strategies such as seeking social support. Future research should explore potential boundary conditions, including personality traits (introverted vs. extraverted), employment sector, and hierarchical position, that can expand our findings. Additionally, incorporating qualitative research methodologies can offer a more nuanced understanding of managers’ underlying logic behind their self-preserving strategies. By investigating the sequence and rationale behind task prioritization during times of emotional exhaustion, qualitative insights can complement quantitative findings, providing a holistic perspective on the dynamics of managerial coping strategies and their impact on organizational change processes. This approach extends beyond solely examining the outcome (i.e., teams’ perception of *laissez-faire*) and delves into the managers’ subjective experiences and decision-making processes.

Finally, considering the cross-sectional nature of our data, we cannot infer any type of causation relationships. To truly understand the sequence in which these variables influence each other, future studies should consider adopting a longitudinal research design. In addition, the means for emotional exhaustion and perception of laisser-faire leadership were rather low. This could imply that our data has devoted managers or that there is a social desirability factor that was not controlled for in our model. Future research could integrate a social desirability scale to control for such a factor. Although team level analysis presents lower risk of the common method bias, our results must be interpreted with cautious because of the cross-sectional nature of our survey.

## 7 Conclusion

In conclusion, our study contributes valuable insights to the understanding of organizational change dynamics, particularly focusing on the crossover effect of managers’ psychological resource scarcity on teams’ collective attitude towards change. Indeed, the distal relationship between a manager’s emotional exhaustion and their team’s readiness to change through perceived laisser-faire and psychological safety provides more nuance to the discussion regarding change management. Managers are often perceived as change agents impervious to the increased demands associated with change management. Our study highlights the importance of considering managers as stakeholders who are also impacted by the organizational change and for whom their resources can become threatened. Supporting overwhelmed managers instead of blaming them for the lack of readiness of their team members could help organizations navigate through organizational change in a more constructive and humane way. Overall, organizations should conceptualize and operationalize their change management using a multilevel logic to care for all stakeholders involved in the organizational change.

## Data availability statement

The datasets presented in this article are not readily available because of contractual obligations but are available from the corresponding author on reasonable request. Requests to access the datasets should be directed to KJ, kevin.johnson@hec.ca.

## Ethics statement

The studies involving humans were approved by Research Ethics Board, HEC Montreal. The studies were conducted in accordance with the local legislation and institutional requirements. Participants were informed of the research and ethical considerations on the first page of our survey, which mentioned that by answering the survey, they consent to our study. This was verified and accepted by the ethics committee of HEC Montréal.

## Author contributions

PG: Conceptualization, Data curation, Formal analysis, Methodology, Project administration, Writing – original draft, Writing – review & editing. FM: Writing – original draft, Writing – review & editing. J-FH: Writing – review & editing. KJ: Conceptualization, Project administration, Supervision, Writing – review & editing.
